# Return for prenatal care and childbirth services among Nigerian women using primary health care facilities

**DOI:** 10.1002/nop2.314

**Published:** 2019-09-19

**Authors:** Joel Ojo Aluko, Regis Rugira Marie Modeste, Oluyinka Adejumo, Rhoda Anthea

**Affiliations:** ^1^ School of Nursing, Faculty of Community and Health Sciences University of Western Cape Cape Town South Africa; ^2^ Department of Nursing Sciences, Faculty of Health and Wellness Cape Peninsula University of Technology Cape Town South Africa; ^3^ Department of Physiotherapy, Faculty of Community and Health Sciences University of the Western Cape Cape Town South Africa

**Keywords:** childbirth, healthcare facilities, prenatal, return, services, women

## Abstract

**Aim:**

The study assessed the return for prenatal care and childbirth services among Nigerian women using primary health care facilities.

**Design:**

A descriptive cross‐sectional approach was employed for the study.

**Methods:**

A total of 730 participants randomly recruited systematically from 21 purposively selected primary health care facilities in Ibadan, Nigeria were studied. A questionnaire and a checklist were used for data collection. The collection of data spanned three months (April to June, 2014). The data were analysed descriptively and inferentially while the results were presented in frequency tables.

**Results:**

The women's mean age was 28 ± 5.3 years. Out of the 730 women studied, 92.6% received prenatal care. The mean difference between the number of prenatal care registration and the number of childbirths was 76.5. Poor environmental hygiene of facilities, statistically significant cost of services and non‐availability of 24‐hr service were implicated for dissatisfaction with care received by the women and consequent poor return rate for childbirth.

## 1 INTRODUCTION

Commonly, women from low socio‐economic background are vulnerable to pregnancy‐related complications (Iliyasu, Abubakar, Galadanci, & Aliyu, 2010; Obiechina, Okolie, Eleje, Okechukwu, & Anemeje, 2011). The peculiarity of their vulnerability predisposes them to finding quicker and cheaper avenues to seek health care. The primary health care (PHC) maternity facilities are to serve this large population of women and their newborns. Services in Nigerian PHC facilities are not completely free, and the costs of available services are relatively determined by staff working in the facilities. Besides, focused antenatal care has not been implemented in almost all the PHC facilities. Thus, there is still tenacious adherence to traditional model of antenatal care requiring multiple clinic visits (Aluko & Oluwatosin, 2008). Reasons for this require inquiry by researchers. If the PHC facilities and services are unsatisfactory to the women, they tend to use informal birthing centres instead of the formal healthcare facilities (Babalola & Fatusi, 2009). Most childbirth undertaken in the informal birthing centres are conducted by untrained attendants. Consequently, such births are characterized by higher perinatal mortality than those undertaken in hospitals or PHC facilities (Owolabi et al., 2008; Ziraba, Madise, Mills, Kyobutungi, & Ezeh, 2009). The status of infrastructures and quality of care available in maternity facilities are likely to determine whether women will return to such health facilities for childbirth services. Based on the foregoing, the women's decision to either return or not return to formal maternity centres, where accessed prenatal care may directly or indirectly link to the outcome of pregnancy (Ziraba et al., 2009).

The quality of places of prenatal care and childbirth services is a product of whether the women are able to access skilled birth attendants or not in such places (Lee, Holden, & Ayers, 2016; Seljeskog, Sundby, & Chimango, 2007). The choice of place of accessing prenatal and childbirth care seems to be critical to neonatal and maternal pregnancy outcomes (Seljeskog et al., 2007). This is so because fewer pregnant women have been observed not to return to the place of their initial antenatal registration for childbirth. This is why the safety of choice of home births and the women's right to make such a choice, when hospital birth is an available safer option still remains controversial issue in developing countries. Although the undesirable outcomes of home delivery such as high maternal and perinatal mortalities or morbidities in developing countries like Nigeria are well‐documented, the success of planned home birth undertaken by unskilled birth attendants have not been reported in the literature (Janssen et al., 2009).

Globally, there were 2.6 million stillbirths in 2009 with over 8,200 deaths a day. At least half of all stillbirths occurred in the intrapartum period. Out of 133 million live babies each year, 2.8 million die in the first week of life. The patterns of neonatal deaths are like the patterns for maternal deaths; most occurring in developing countries (World Health Organization (WHO), 2013). Up till now, the highest burden of the yearly maternal and neonatal morbidities and mortalities resulting from pregnancy‐related complications worldwide is in the developing countries. With reference to Nigeria, the unacceptable complications are evident in the 2013 final report of the Nigeria Demographic Health Survey (NDHS) where the reported estimated Maternal Mortality Ratio (MMR) was 576 per 100,000 live births during the seven‐year period preceding the survey (National Population Commission (NPC), 2014). This implies that for every 1,000 live births in Nigeria during the seven years preceding 2013, approximately six women died during pregnancy or within two years of childbirth (NPC, 2014). The NDHS 2013 put the lifetime risk of maternal death at 0.033, indicating that about 3% of women died during pregnancy, childbirth or within two months of childbirth. The estimated MMR in 2013 (576/100,000 live births) is almost the same as in the 2008 NDHS report (545/100,000 live births) (NPC, 2014). Similarly, Nigeria's newborn death rate (neonatal mortality) is one of the highest in the world and has been documented to be 528 per day (Ibeh, 2008). Thus, Nigeria contributes 10% to the global mortality rate (Ibeh, 2008; NPC, 2014; WHO, 2012). Research has shown that most of these deaths could be prevented, if women have access to skilled care throughout pregnancy, childbirth and the postpartum period (WHO, 2006).

Varying qualities of health care in different available settings are rendered to women and their newborns. This is an issue of public and reproductive health concern to stakeholders. Poor quality of health care is a major factor contributing to the high maternal, neonatal and child mortality rate in sub‐Saharan Africa, particularly Nigeria (Choudhry, 2005). In Nigeria, birthing centres fall into two categories, namely informal and formal birthing centres (Aluko & Oluwatosin, 2008). The informal birthing centres are operated by unskilled birth attendants who have no midwifery training and are therefore not licensed by the Nursing and Midwifery Council of Nigeria (NMCN). These birth attendants are found operating traditional birth attendant centres, faith‐based mission homes owned mostly by Christian religious churches. The formal birthing centres are the centres owned by the governments, private individuals or corporate organizations. The major birth attendants are qualified medical or nurse/midwife professionals who have been licensed to undertake maternity care services by the Nigerian Medical Association (NMA) and the NMCN, respectively. Usually, women are encouraged to use formal health facilities for prenatal, natal and postnatal care to avoid the undesirable outcomes associated with pregnancy and childbirth undertaken in informal alternative birthing centres. However, it is worthwhile to appraise the proportion of the teaming population of vulnerable Nigerian women that have access to prenatal care and childbirth services at primary level. On this premise, the study sought to examine whether Nigerian women return to PHC facilities where they initially accessed prenatal care for childbirth services.

## 2 METHODS

### 2.1 Study design and settings

This clinic‐based study employed a descriptive cross‐sectional design to evaluate the trends of perinatal care in primary healthcare centres and rate of return to the same centres for childbirth care among postnatal women in an urban centre of South‐Western Nigeria. The respondents were recruited across the five Local Government Areas within the Ibadan metropolis. Ibadan is the state capital of Oyo state, Nigeria and the largest city in West Africa sub‐region. The city is densely populated with most of the population living in slums and high‐density areas.

### 2.2 Instrument and data collection

The study used a validated self‐administered structured questionnaire and a checklist for data collection. The questionnaire, which was designed in line with WHO's “Assessment tool for quality of hospital care for mothers and newborn babies” has four sections (A–D). The 5‐item Section A elicited the participants' socio‐demographic data, while section B having 17 items elicited participants' obstetric history. Section C assessed participants' experiences at the service centres (WHO, 2010). Thus, the questionnaire was adapted from that of the WHO. The study focused primarily on assessment of return rate for prenatal and childbirth services among Nigerian women. Additionally, section D focused on health workers' attitudes and supports as well as clients' satisfaction with the care they received from at the facilities were examined. The local language (Yoruba language) version of the questionnaire was produced using “back‐to‐back” translation and thus was made available to women who could not comprehend English language version. The reliability coefficient of the research tool was 0.8. Thus, it was considered adequate for the study (Creswell & Clark, 2007). In addition, the checklist was used to record the population of women who registered for prenatal care, women who have childbirth care and children brought for vaccination in each of the PHC facilities.

Women who brought their babies for immunization service were recruited to participate in the study. The data collection procedure took place in the waiting areas of child welfare clinics of the PHC facilities. The data collection was facilitated by the healthcare workers in each of the facilities. Data collection spanned four months, and it took 25–30 min for each participant to complete the questionnaire.

### 2.3 Population and sampling technique

A total of 21 purposively selected PHC facilities within the five Local Government Areas (LGAs) in the urban city. The PHC facilities were purposively selected for the study because they were nearer to women from low socio‐economic background and thus vulnerable. The study population were postnatal women who brought their newborns to the PHC centres for immunization against childhood communicable diseases.

The sample size for the surveys was based on Stoker's (1985) table titled “sampling in the quantitative paradigm” (Van Griensven, Moore, & Hall, 2014). This was adopted, being more empirical and easy to adapt for survey studies (Van Griensven et al., 2014). In line with the Stroker's table, the percentage samples recommended for different ranges of population were used for selection of appropriate sample size for PHC centres and the respondents. Therefore, for the estimated total population of 13,437 respondents, 4.5% was recommended by Stoker (Van Griensven et al., 2014). Hence, in this study, 4.5% of 13,437 were calculated to be 604 respondents. In addition, 25% of 604, which was equal to 151 was added for attrition rate. Thus, the sum of 604 samples plus 151 attrition rate amounted to 755 respondents used for this study.

A systematic random sampling technique was used to recruit the 755 postnatal women (clients) from the estimated population of 13,437. The attendance records served as sample frames for the postnatal women. The sample intervals were calculated for the population using the statistical formula: *K* = *N*/*n*, where *K* = Sample interval; *N* = Total population in the sample frame; *n* = sample size (Daniel & Cross, 2010). The sample frame of each PHC centre was used to compute the sample interval.

## 3 INCLUSION AND EXCLUSION CRITERIA

These were women whose babies were within day one day 42 or 6 weeks after childbirth, who attended child welfare/immunization clinics and were willing to participate in the study. The women with newborns aged between 1–42 days were included in this study to capture the range of services they were exposed to from pregnancy till puerperium. Besides, it was believed that most of those women would be able to remember nearly all their experiences within six weeks of childbirth. Women who were either not willing or too ill following childbirth were excluded from the study.

### 3.1 Statistical analytical methods

The population of the prenatal women and the babies was derived from the antenatal and child immunization attendance registers of each PHC facility, respectively. Descriptive and inferential statistics were used for data analysis with the aid of the Statistical Package of Social Sciences (SPSS) version‐21. The ages of the women were classified into teenage, matured and elderly mothers. The classification was based on the associated relative health risks of each category. Paired *t* tests were performed to establish the degree of statistically significant differences among quality ratings of facilities by participants, between populations of prenatal women versus women who returned to the PHC facilities under study and population of babies immunized versus childbirth undertaken at the PHC facilities under study. Level of significance was reported at 5% probability level.

## 4 RESULTS

### 4.1 Socio‐demographic and obstetric characteristics of the participants

Out of the 755 questionnaires administered 730 were retrieved and analysed. Hence, the response rate was 96.7%. The women's mean age ± standard deviation was 28 (*SD* 5.3). Table 1 presents the socio‐demographic characteristics of the participants.

**Table 1:** Socio‐demographic characteristics of participants (Mean age = 28 ± 5.3)

Variables	Overall^a^	Category A^b^	Category B^c^
Age group			
Teenage mothers	20 (2.7%)	7 (2.5%)	13 (2.9%)
Mature mothers	604 (82.7%)	231 (83.1%)	373 (82.5%)
Elderly mothers	106 (14.5%)	40 (14.4%)	66 (14.6%)
Marital status			
Without partner	58 (7.9%)	25 (9.0%)	33 (7.3%)
With partner	672 (92.1%)	253 (91.0%)	419 (92.7%)
Level of education			
Informal	10 (1.4%)	6 (2.2%)	4 (0.9%)
Primary	113 (15.5%)	50 (18.0%)	63 (13.9%)
Secondary	432 (59.2%)	175 (62.9%)	257 (56.9%)
Postsecondary	37 (5.1%)	11 (4.0%)	26 (5.8%)
Tertiary	138 (18.9%)	36 (12.9%)	102 (22.6%)
Religion			
Christians	359 (49.2%)	94 (33.8%)	265 (58.6%)
Muslims	371 (50.8%)	184 (66.2%)	187 (41.4%)
Occupation			
Unemployed	67 (9.2%)	22 (7.9%)	45 (10.0%)
Students/National Youth Service Corps	51 (7.0%)	22 (7.9%)	29 (6.4%)
Employed	612 (83.8%)	234 (84.8%)	378 (83.6%)

a The total of women in category A and B was 730 women.

b 278 Women who received prenatal care at the setting where data were collected.

c 452 Women who received prenatal care from other facilities but brought their babies to the setting where data were collected for immunization.

Among the women studied, 258 (35.3%) of them came to the PHC facilities with their first babies while the remaining came with subsequent babies. Specifically, 442 (60.5%) of the women had between one and two children, 257 (35.2%) had 3–4 children, while 31 (4.2%) had more than 4 children.

### 4.2 Places of prenatal and childbirth care

Out of the 730 women studied, 676 (92.6%) received prenatal care. The remaining 54 (7.4%) women gave various reasons for not accessing prenatal care. The given reasons are shown in Table 2. Table 3 shows the various healthcare facilities where the 676 women received prenatal care.

**Table 2:** Reasons given by participants for non‐use of prenatal care

Reasons for non‐use of prenatal services	*N*	%
Lack of accessibility	27	50.0
Dislike for service provision	7	13.0
Too expensive	6	11.1
Incompetent health workers	4	7.4
Financial constraint	6	11.1
The pregnancy was unwanted	4	7.4
Total	54	100.0

**Table 3:** Healthcare facilities where participants received prenatal care

Facilities	*N*	%
TBA centre	15	2.2
Faith‐based clinics	78	11.5
Private hospitals/clinics	145	21.4
PHC centre	199	29.4
Formal mission hospitals	72	10.7
State/Federal hospitals	167	24.7
Total	676	100.0

Out of the 676 women who received prenatal care, 153 (22.6%) registered for prenatal care in two healthcare facilities, while the remaining 523 (77.4%) registered in one facility throughout the entire periods of pregnancy. Those who registered for prenatal care in two healthcare facilities gave various reasons for their actions, which include comparative distances, costs, attitudes of health workers, availability of 24‐hr maternity services, access to spiritual care in their two chosen health facilities and fear of developing complications that might require emergency obstetric care (Table 4).

**Table 4:** Reasons for booking for prenatal care in two different health facilities

Reasons for booking in two health facilities	*N*	%
No reasons given for booking in two facilities	77	50.3
Because I reside in two places	5	3.3
Because of incessant strike in government facilities	3	1.9
Disrespectful treatment from health workers in formal centres	1	0.7
Fear of falling into labour at night	3	1.9
In anticipation of emergencies	6	3.9
Just to have access to Tetanus injection	2	1.3
Long waiting time in formal centres	1	0.7
Nearness of the alternative centre	18	11.8
No provision of 24‐hr childbirth service in the formal centre	3	1.9
To avoid expensive cost of services in formal centre	2	1.3
To get proper care in formal centre	15	9.9
To have access to both prayer in faith‐based & medical care in formal centre	17	11.1
Total	730	100.0

Furthermore, not all the participants returned to the same healthcare facilities used for prenatal care initially for childbirth. Among the 278 women who received prenatal care in the settings where the data were collected (i.e., women in category A), 72 (25.9%) did not return to the same setting where the data were collected for childbirth. In addition, out of the 206 women who returned to the settings where the data were collected for childbirth, 118 (42.4%) preferred another healthcare facility to the same PHC facilities where they had their last childbirth care. Besides, they would not recommend PHC facilities where the data were collected to other women for prenatal and childbirth services. In contrast, among the 452 women who did not received prenatal care at the PHC facilities where the data were collected but brought their children to the same PHC facilities for only immunization services (i.e., women in category B), 191 (42.3%) returned to the various healthcare facilities where they had initially received prenatal care for childbirth. A total of 84 (18.6%) did not return to the various healthcare facilities where they accessed prenatal care for childbirth. Thus, this category of women gave birth to their babies in other healthcare facilities other than the initial place of prenatal care. The category of women (amounting to 25.4%) while comparing their place of childbirth and the PHC where the data were collected (the place where they brought their children for immunization) recommended their choice of place of childbirth rather than the PHC facilities where the data were collected (i.e., where they brought their children for immunization as at the time of data collection) to other women.

One hundred and eighteen (60.5%) of women in category A recommended the place of their last childbirth to other women as against 115 (40.1%) who did not. Those in the second group form category B. On the other hand, 77 (39.5%) and 172 (59.9%) of women in category A and B, respectively, recommended the settings where data were collected to other women. The various factors influencing the choice of their places of childbirth are presented in Table 5.

**Table 5:** Factors influencing choice of place of childbirth among participants

Reasons for choices of places of childbirth	Category A^a^		Category B^b^	
	*N*	%	*N*	%
The facility is nearer	64	23	109	24.1
The services in that facility is better	112	40.3	34	7.5
The services there are less expensive	21	7.6	21	4.6
The health workers there are more competent	60	21.6	25	5.5
Fewer delivery materials are demanded	35	12.6	15	3.3
I didn't care because the pregnancy was unwanted	9	3.2	10	2.2
The Health Workers are more friendly and respectful	64	23	47	10.4
The Health workers take care of my concern more seriously	80	28.8	37	8.2
Total	445	100	298	100

a Category A: Women who received prenatal care and/or childbirth services at the settings where data were collected.

b Category B: Women who received prenatal care in various health facilities other than the settings where data were collected but utilized the settings where data were collected for child immunization services.

The women who received childbirth care in various healthcare facilities other than the PHC facilities where data were collected but brought their babies to the later for immunization only (women in category B) rated the two facilities (i.e., the former and the later) on a five‐point scale. The six aspects of the facilities that were rated using the five‐point scale included environmental hygiene, labour wards, toilets, bathrooms, building appearance and staff attitude. In the rating, the women considered the status of the various health facilities (they considered whether where they gave birth to their babies were better than the places where they were accessing immunization services, that is the PHC facilities where data were collected). The difference was found to be statistically significant (Table 6).

**Table 6:** Quality of PHC facilities under study versus other health facilities used by the women

A. Facility rating score difference Quality rating of other health facilities versus quality rating of the settings where data were collected							
Statistics	Paired samples	*N*	Mean	Std. deviation	Std. error mean	*t*‐statistic	*p*‐value
Paired *t* test	Rating of other Health Care facilities by category B women	452	21.89	6.70	0.32	−8.32	0.001*
	Rating of the settings where data were collected by category B women	452	18.97	6.02	0.28		

B. Facility rating score difference Quality rating of the settings where data were collected versus other health facilities							
	Independent samples	*N*	Mean	Std. deviation	Std. error mean	*t*‐statistic	*p*‐value
Independent *t* test	Rating of other Health Care facilities by category B women	452	1.89	6.70	0.32	−1.77	0.08
	Rating of the PHC where data were collected by category A women	278	2.72	4.96	0.30		

C. Population difference Number of women who registered for prenatal care versus number of women who returned for childbirth at the PHC where data were collected							
	Paired samples	*N*	Mean	Std. deviation	Std. error mean	*t*‐statistic	*p*‐value
Paired *t* test	Number of women who registered for prenatal care at the PHC where data were collected	21	141.86	105.0	22.91	5.95	0.001*
	Number of women who returned for childbirths at the PHC where data were collected	21	65.38	63.33	13.82		

D. Population difference Number of babies immunized versus number of recorded childbirths at the PHC where data were collected							
	Paired samples	*N*	Mean	Std. deviation	Std. error mean	*t*‐statistic	*p*‐value
Paired *t* test	Number of babies immunized with BCG at the PHC where data were collected	21	254.62	139.84	30.51	8.06	0.001*
	Number of Childbirths undertaken at the PHC where data were collected	21	65.38	63.33	13.82		

Note

*N* = population sample, *p*‐value = level of significance.

* Statistically significant.

Similarly, the settings where data were collected (the places where the 278 women accessed prenatal and childbirth care but was used by all the participants for child immunization) were rated less than other healthcare facilities (the places where 452 women gave birth to their babies). The difference was found to be statistically significant (Table 6).

There was a statistically significant difference between the mean attendance population of women using antenatal services and that of women returning to the PHC centres for childbirth, *p*‐value < 0.05 (Table 6). This implies that the women who returned to the PHC centres for childbirth were fewer in number than women who received prenatal care in the same centres. Similarly, there was a statistically significant difference between the mean attendance population of women who brought their babies for immunization services and that of women who received childbirth services in the PHC centres, *p*‐value < 0.05 (Table 6). This implies that women who use other birthing centres (homes, TBA centres, mission homes, private hospitals/clinics, other government hospitals and maternity centres) but converged at same PHC centres for child immunization were more in number than women who gave birth to their babies in the same PHC centres.

## 5 DISCUSSION

In the overall, most participants were mature women of childbearing age ranging between 20–34 years while the teenage mothers among the participants were few. That was probably for lack of necessary social supports to attend formal healthcare facilities. Most previous studies conducted with either pregnant women or nursing mothers as participants had reported similar findings (Aluko & Oluwatosin, 2008; Caughey, Cahill, Guise, & Rouse, 2014; Strydom, 2011). Similarly, the reported larger population (92.1%) of married women living with their partners is a common trend in Nigeria (Adeyinka et al., 2010; Kruk et al., 2009; Oluwatosin, Aluko, & Onibokun, 2011). Furthermore, it was observed that less than 20 per cent of the women had lower than secondary school education. This is quite a good development, as this shows that more Nigerian women are becoming educated. This positive development can improve the health behaviour of the women. In addition, the employment status observed in the study may not necessarily translate into financial empowerment, particularly with those who were self‐employed and those who worked with private institutions.

Most of the women were either mothers who had experienced childbirth once (primiparae) or mothers who had experienced childbirth more than once (multiparae). Similarly, those who had between one child and four children were in the majority. This health behaviour conforms to the modern family planning advocacy and practice in Nigeria as against what it used to be in the past decades. The findings of the study confirmed that about 90% of the 730 women studied received prenatal care. Among those who received prenatal care, 22% registered in more than one health facility for various reasons such as proximity of the facilities to their residences, cost implication of care, negative attitude of health workers, unavailability of 24‐hr services in some centres, availability of spiritual care in the faith‐based centres and fear of developing complications that may not be properly handled in some facilities than their more preferred choices. Those who did not register for prenatal care gave similar reasons for their action. These findings concur with that of Onah, Ikeako, and Iloabachie (2006), where women gave reasons for their choice of place of prenatal care and childbirth. Although the women gave reasons akin to those given by the Enugu women for choosing two places for prenatal care, the reported antenatal registration in two different centres was likely to be a sign of indecision about their more preferred place of birth (Creswell & Clark, 2007).

The findings equally revealed that the women rated the other places of childbirth higher than the PHC facilities under study. This implies that most of the women came primarily to the PHC setting for child immunization. This is because other places of childbirth, such as the faith‐based centres, traditional birth attendant (TBA) centres and private hospitals do not usually offer immunization services to clients. Onah et al. (2006) reported increased use of prenatal care among women of Enugu in the South‐Eastern part of Nigeria.

From the findings, it is apparent that women exercise their legitimate rights in choosing facilities they want for either their prenatal or childbirth care. This expected behaviour calls for some considerations because it has been argued that patients do not know what the professionally acceptable level of care is (Kaim‐Caudle & Marsh, 1975). For instance, in the study conducted by Ehiri, Oyo‐Ita, Anyanwu, Meremikwu, and Ikpeme (2005) among women in Calabar, South‐Southern part of Nigeria, many of the mothers perceived the quality of care they received as satisfactory, while shortage of medications, lack of preparedness for emergencies and long waiting hours were common complaints. It has been argued that dissatisfaction is an indication that services delivered are lacking in some aspects (Rashid & Jusoff, 2009; Rosenthal & Shannon, 1997; Weiss & Rose, 1988). Besides, lack of medications and long waiting hours have been shown to contribute to poor use of services (Katung, 2001; Sauerborn, Nougtara, & Diesfeld, 1989). Therefore, all facilities providing maternity care are to be monitored for compliance with respect to the expected standard of care rendered to women. Rather than discouraging women from receiving care from one facility or the other, improving maternity services in various facilities will likely yield a better outcome.

From the results of this study, it was observed that among the 278 who received prenatal care in the PHC facilities under study, more than one‐quarter eventually did not return there for labour and childbirth. Again, more than 40 per cent of those who returned to the PHC facilities, where they had their prenatal care decided not to return to the same centres in subsequent pregnancies. Besides, these women would not be willing to recommend to other women the PHC centres where they had their current babies. Mpembeni et al. (2007) reported a similar finding in their study among Tanzanian mothers where only 46.7% of the women who booked for antenatal care in health facilities returned for childbirth. However, since the current study is institution‐based, it may not be possible to have reported accurately the percentage of home deliveries. These factors described as reasons for dissatisfaction require a serious attention by stakeholders, because the availability of appropriate infrastructures with hygienic toilets and bathrooms will contribute to therapeutic milieu of the women (McLachlan, Forster, Yelland, Rayner, & Lumley, 2008).

The study showed the results of the rating of the health facilities the women used for prenatal and childbirth care. The rating was done on a 5‐point Likert scale. The aspects rated by the women included the following: environmental hygiene, labour ward, toilet, bathroom, building appearance and staff attitude. Other healthcare facilities were rated significantly higher than the PHC facilities under study by those who accessed prenatal and childbirth care in other healthcare facilities. The report of the rating further confirmed that most of the PHC facilities require complete overhaul in respect of the aspects considered in the rating (i.e., environmental hygiene, labour ward, toilet, bathroom, building appearance and staff attitude).

In addition, the study revealed various expressions of dissatisfaction with different aspects of the maternity services by the women. Those aspects included the following: type of services, condition of building infrastructure, inadequate equipment and medications, attitudes and incompetence of the health workers. All these require prompt attention of the stakeholders.

Apart from that, a situation where pregnant women or postnatal women found no waiting space is dehumanizing and tantamount to abuse of their right to respectful maternal care (Bowser & Hill, 2010). Every woman has the right to be treated with dignity and respect. No one should humiliate or verbally abuse a woman for any reason. Service providers must ensure that women are as comfortable as possible during procedures (Aluko, 2015; Bowser & Hill, 2010). Similarly, it is very discomforting and not dignifying for a woman who has newly been delivered of a baby not to have a place she could empty her bowel, empty her bladder or take her bath despite the usual soiling from vaginal fluid/blood that commonly characterizes labour and childbirth. This unacceptable condition of most of the PHC facilities requires immediate intervention from the appropriate stakeholders. It is a form of denial of human right to dignifying health care because it makes them uncomfortable. Therefore, it must be discouraged (Aluko, 2015; Bowser & Hill, 2010). Consequently, more spacious pieces of land should be sought from the community whenever PHC facilities that are meant to provide maternity services are being considered. This can improve clients' satisfaction. The various evidences of patient dissatisfaction with the quality of maternity care services that were offered to them in the PHC facilities have been implicated for reduction in use and non‐use of formal public healthcare facilities (Phellas, Bloch, & Seale, 2011).

The findings of this study show that the population of women who eventually gave birth to their babies in the studied PHC settings were significantly fewer than those who commenced antenatal care in the centres. Similarly, the population of mothers who brought their babies for immunization was significantly more than the number of mothers who delivered their babies at the PHC facilities. This implies that women who use other health facilities for prenatal care and/or childbirth care converged in the PHC centres for child immunization. The findings suggest that the PHC facilities require attention and intervention.

### 5.1 Limitation

Only PHC facilities providing maternity services that are considered as “viable” by management of the LGAs were used for this study. Other facilities that were not designed for maternity services and those that had no regular gynaecological and obstetric patients were excluded based on the recommendations, management of the LGAs.

### 5.2 Conclusion

From the study, a statistically significant percentage of the women who received prenatal care in the PHC facilities did not return there for childbirth. Similarly, many women who received prenatal care and childbirth care in the PHC facilities would prefer to receive both prenatal and childbirth care in other health facilities during subsequent pregnancies because they were not satisfied with the care received in their last pregnancies. Moreover, the population of women who registered for prenatal care in more than one place was quite large while those who used faith‐based healthcare facilities did so to access spiritual care. It might be very difficult to influence them to do otherwise. Besides, the quality rating of the PHC facilities was significantly less than that of the other healthcare facilities. Therefore, the renovation of existing structures and the building of new ones are recommended. In addition, all other dimensions contributing to good quality maternity care services in all other facilities should be evaluated, restructured and monitored for attainment of an acceptable level of quality.

## CONFLICT OF INTEREST

The authors declare no competing interests.

## AUTHORS' CONTRIBUTIONS

AJO initiated and conducted the study, analysed the data and wrote major parts of the manuscript. AR supervised the study and wrote statistically significant parts of the manuscript. She reviewed and edited the manuscript for final submission. MRRM supervised study and wrote statistically significant parts of the manuscript. She reviewed and edited the manuscript for final submission. All authors read and approved the final manuscript. AO supervised the study proposal in preparation for fieldwork. He reviewed and edited the research tools as well as the manuscript.

## ETHICAL APPROVAL

The study protocol was approved by the Senate of University of the Western Cape, Bellville, South Africa (Case reference numbers: 13/10/23), and Oyo State Ethical Committee in Nigeria (Case reference numbers: A3/479/576). Participants were recruited into the study after written‐informed consent had been obtained from each of them. Participation was made voluntary. Thus, participants were neither deceived nor coerced to participate in the study.

## DATA AVAILABILITY

The datasets used and/or analysed during the current study available from the corresponding author on reasonable request.
